# Device Closure of Perimembranous Ventricular Septal Defect: Choosing Between Amplatzer Occluders

**DOI:** 10.3389/fped.2019.00300

**Published:** 2019-08-16

**Authors:** Raymond N. Haddad, Linda Daou, Zakhia Saliba

**Affiliations:** ^1^Department of Pediatrics, Hotel Dieu de France University Medical Center, Saint Joseph University, Beirut, Lebanon; ^2^Department of Pediatric Cardiology, Hotel Dieu de France University Medical Center, Saint Joseph University, Beirut, Lebanon

**Keywords:** ventricular septal defect, perimembranous, amplatzer, device closure, complete heart block

## Abstract

**Background:** Off-label device closure of perimembranous ventricular septal defect (pmVSD) is well reported in the literature with encouraging results. However, technical challenges may be encountered.

**Objectives:** To evaluate and compare feasibility, technical aspects, procedural outcomes, and mid-term follow-up of pmVSD closure using Amplatzer™ occluders.

**Patients and Methods:** From July 2015 to July 2018, patients in whom pmVSD closure was attempted using an Amplatzer occluder were retrospectively identified from our institution's database. Device selection was made according to the defect anatomy that was obtained via ventriculography and trans-esophageal echocardiography. Follow-up evaluations were done at discharge, then at 1, 3, 6, and 12 months and yearly thereafter with transthoracic echocardiography and electrocardiogram.

**Results:** In total, 8 Amplatzer Duct Occluder (ADO), 27 ADO II, and 17 Amplatzer Muscular VSD Occluder (AMO) were used in 51 patients with a mean age of 7.4 ± 6.9 years and a mean weight of 25.4 ± 19.8 kg. Implantation was successful in 50/51 patients (98.0%). There was no procedure related mortality. One ADO accidentally embolized to the aorta after release and was surgically recaptured from the iliac artery. All ADO II were delivered retrogradely with the least amount of time (*p* = 0.002) and the lowest radiation exposure (*p* < 0.001). Minor valvular disturbances occurred in 8/49 patients (16.3%), including five tricuspid regurgitation (three with ADOII and two with AMO) and three trivial aortic regurgitations (two with ADO and one with ADOII). On a median follow-up of 194 days (range, 60–895 days), no surgical device removal was necessary. At 6 months of follow-up, trivial residual shunt was present in 5/49 patients (10.2%), among which none occurred with ADO. One complete atrioventricular block was detected 18 months after ADO implantation and required permanent pacing.

**Conclusions:** Transcatheter closure of PmVSD using Amplatzer occluders is feasible, safe and efficacious in properly selected patients. The major key factor behind high procedural success rate is proper device selection. ADOII is remarkably superior in terms of device softness, flexibility and faster implantation process. Yet, its use is limited to small defects with particular anatomy.

## Introduction

Ventricular septal defect (VSD) is the most common congenital heart disease (CHD) with the perimembranous VSD (pmVSD) being the most common subtype (1–3). While spontaneous closure rates are high, surgical repair may be indicated during early infancy in case of severe pulmonary hypertension, or failure to thrive despite optimal medical management (3). Later in life, an unknown percentage of patients with small residual defect develop cardiac problems, and then become candidates for closure. Due to advances in cardiac imaging modalities and techniques, interventional pmVSD closure has become increasingly acceptable with the availability of different occlusion systems but it remains technically challenging (4, 5). When compared to surgery, percutaneous approach avoids sternotomy and has the potential advantages of lower morbidity, faster recovery, shorter hospital stay, and reduced costs (6–8). Ideally, device closure would be easily handled with low rates of residual shunt (RS) and a special attention to the aortic and tricuspid valves while avoiding the conducting tissues. The first serious device designed for pmVSD closure was conceived by Amplatzer in the late 90's and had an asymmetrical design (9). However, high incidences of complete atrioventricular block (CAVB) led to abundance of this device with a continuous search for a better substitute (10–12). Therefore, published literature reported successful use of devices that were originally conceived for other defects (4, 5). The aim of this study is to review our transcatheter pmVSD closure experience using different Amplatzer™ devices (Abbott, USA) and to evaluate the midterm follow-up outcomes.

## Patients and Methods

### Study Population

This is a retrospective monocentric study. The records of all patients with a hemodynamically significant pmVSD and scheduled for attempted closure using an Amplatzer^TM^ occluder between July 2015 and July 2018 at the Saint Joseph university teaching hospital, Hotel Dieu de France, were reviewed and included in this study. Permission was obtained from the company to use and mention their product in this submission. Data were collected from first admission until last available follow-up. Patients' demographic information, cardiac diagnosis, procedural data, complications, size and type of the duct occluder devices, re-interventions needed, and procedural outcomes were collected from the medical records. Study protocol was reviewed and approved by the institutional review board.

Prior to cardiac catheterization, 2D transthoracic echocardiography (TTE) was performed to all patients by an experienced operator, with a GE Vivid 3 machine including M mode, two-dimensional and Doppler examination. Size and shape of VSD were determined by standard four-chamber view. Sub-aortic rim (SAR) was defined by the distance between the upper margin of the defect and the aortic valve (AoV) and was evaluated using the five-chamber view and parasternal long axis view (PLAV). Parasternal short axis view was used to identify the defect position on an analog clock, number and diameters of the right ventricle (RV) exit(s) as well as LV entry diameter. These echocardiographic measurements guided the selection of the device size which was later reassessed, intraoperatively, by angiography & transesophageal echocardiography (TEE).

#### Inclusion Criteria

For the purpose of this study, pmVSD with indication for transcatheter closure was defined by clinical or TTE evidence of a significant left to right shunt due to isolated pmVSD, with the presence of at least one of the following criteria: (1) estimated pulmonary-to-systemic blood flow ratio (Qp/Qs) > 1.5; (2) prominent cardiomegaly, defined as cardiothoracic ratio >0.55 on standard chest X-ray (CXR); (3) left atrial (LA) enlargement, defined as a LA-to-aortic diameter ratio >1.5 on the PALV examination; (4) left ventricle (LV) overload and enlargement, defined as LV end-diastolic z-score on echocardiogram, indexed to body surface area ≥2.0; (5) history of infective endocarditis related to the pmVSD; and (6) symptoms, including recurrent respiratory infections (defined as ≥6 events in the preceding 12 months) and/or failure to thrive.

#### Exclusion Criteria

Patients considered not eligible for the procedure had one or more of the following anatomical or clinical criteria: (1) pmVSD with a prolapse aortic cusp, aortic regurgitation (AR) or aortic valve stenosis, infundibular defect, septal mal-alignment, SAR ≤ 1 mm (in non-aneurysmal anatomy); (2) severe pulmonary artery (PA) hypertension and a right-to-left shunt (unless PA banding or congenital pulmonary valve stenosis) or pulmonary vascular resistance > 8 Wood units or documented irreversible pulmonary vascular disease; (3) presence of any other associated CHD unrepairable percutaneously; (4) active bacterial infections or endocarditis or sepsis (local/generalized); (5) contraindication to antiplatelet or anticoagulation therapy or agents; (6) and a body weight <8 kg. Preoperative routine examination including standard 12 leads electrocardiogram (EKG), CXR, TTE, and blood test were performed on all patients.

### Interventional Procedural Technique

Written informed consent was signed by the patients or parents of the children after they were provided with a comprehensive explanation about the procedural details, the advantages and possible complications. All procedures were performed by the same operators, in the catheterization laboratory, under general anesthesia, TEE, and fluoroscopic control. Special attention was given to minimize hypothermia. One femoral vein (FV) and one 5F contralateral arterial line were obtained. After that, intravenous (IV) heparin (100 IU/kg, 5,000 IU maximum) was administered to all patients and was regularly monitored to maintain activated clotting time longer than 200 seconds. Prophylactic antibiotic therapy using IV cefazolin (30 mg/kg, 2,000 mg maximum) was also given at the beginning of the procedure and two subsequent doses (every 8 hours during the following 24 hours). Standard right and left cardiac catheterization were performed and data was gathered. Left ventriculography with a marked pigtail catheter was performed at 55–60° left anterior oblique to 20° cranial projection in order to profile the defect and was combined to intraoperative TEE to accurately determine the pmVSD location, shape, depth, size and its relationship with adjacent aortic and tricuspid valves. The RV defect exit was more clearly evaluated on TEE especially in aneurysmal anatomy. In case of multiple RV exits, the largest one was chosen as a target measurement. The defect entry diameter was measured on angiography at the largest diastolic phase on LV side.

### Device Selection

The three available Amplatzer™ occluders (Abbott, USA) used in this procedure were Amplatzer Duct Occluder (ADO), Amplatzer Duct Occluder II (ADO II), and Amplatzer Muscular VSD Occluder (AMO). Due to its soft and flexible nature design and its fast retrograde deliverability, ADO II was our first choice of selection and it was installed when the following criteria were met: defect diameter <5 mm with an SAR larger than 3 mm in non-aneurysmal type defects. In aneurysmal type, the RV exit should be <5.5 mm with a LV entry diameter <12–12.5 mm but large enough to accept the left disk (LD) in the aneurysm especially when the SAR is <3 mm. The diameter of the device waist in ADO II was chosen to be 1 mm (±0.5 mm) greater than the smallest VSD diameter.

When ADO II device was not applicable, ADO device was favored over the AMO in patients with big aneurysms. The device size was selected so that the right disk (RD) diameter (the pulmonary end) would be 2 mm (±0.5 mm) greater than the smallest VSD diameter, in order for the subsequent left retention skirt diameter (aortic end of the device) to be totally accommodated inside the aneurysm, especially when the defect depth allowed the RD to reach the RV exist. On the other hand, when LD diameter was greater than LV entry diameter, ADO was installed when SAR was longer than 3–3.5 mm with a 7mm maximal VSD depth to allow RD squeezing in the RV exist.

Finally, the AMO device was chosen according to the following protocol: when the SAR was >4.5 mm, the device waist was chosen according to the LV entry diameter without any need to oversize. When the SAR was <4.5 mm, the device waist was chosen equally to the RV diameter of the aneurysm, and was implanted when the subsequent LD fitted entirely in the aneurysmal LV opening. It is noteworthy that in low budget countries' catheterization labs, it is not feasible to keep all range on shelves. For that, device selection was sometimes influenced by device price and availability.

### Delivery

#### Venous Approach

This antegrade approach was used to implant all ADO and AMO. The VSD was crossed retrogradely from the LV side, using a 4 or 5 F Judkins right (JR) coronary catheter (Cordis Corporation, Florida, USA) and 0.035 inch J tip Terumo glide wire (Terumo Corp. Japan) combination. Once across the VSD, the catheter was advanced into either branch of the PA, or preferably into the superior or inferior vena cava. The Terumo wire was replaced with a 300 cm noodle wire (Abbott, USA) that was then snared and exteriorized through FV, using an Amplatz Gooseneck Snare (ev3 Inc.,; Minnesota, USA) to create an arteriovenous circuit (AVC). Over this wire, an appropriate 6, 7, or 8 F Amplatzer 45 or 180° delivery system was advanced from the FV across the VSD all the way until the tip of the sheath arrived to the ascending aorta. The dilator was then removed from the vein line while the guide wire and the end-hole catheter were removed from the arterial line. After flushing the long sheath, the chosen device was loaded under a saline solution and was advanced, without rotation, to the tip of the delivery sheath under fluoroscopy. The distal disk was partially opened in the ascending aorta and then gently pulled back through the AoV into the LV. After that, delivery sheath was slowly retracted until the distal disc was completely deployed at the LV side of the VSD. The entire assembly (delivery cable and delivery sheath) was then pulled back as one unit into the defect and the sheath was retracted to deploy the waist of the device in the VSD. Once the position was confirmed by angiography and TEE, sheath was retracted to deploy the proximal disc. After full deployment of the occluder, TEE combined with LV angiography were performed again to verify the position and the shape of the device, RS and the absence of interference with the AoV cusps. At this point, the delivery cable passing across the TV generated some regurgitation and it was difficult to predict its evolution after device release. The device was then released by turning the cable counterclockwise after confirmation of good device position and absence of AoV disturbances. Final result was only assessed by TEE to avoid angiography, catheter manipulation in the LV and accidental mobilization of the device.

#### Arterial Approach

This retrograde approach was used to implant all ADOII. After crossing the defect from the LV side using the same technique as above, a 5F delivery catheter TorqVue™ LP was introduced from the FA and advanced over an exchange wire, through the VSD. The selected device was loaded in the delivery sheath and advanced to its tip into the RV. The delivery catheter was pulled back slowly under TEE guidance into the RV, near the defect. Once the position was confirmed, the distal disk was slowly advanced out the catheter and all system was pulled back as one unit against the septum. At this stage, the absence of tricuspid regurgitation (TR) related to the RD was confirmed by TEE. The catheter was then retracted to allow the waist and proximal disk to open against the left side of the septum with gentle tension. The device position was then assessed using TEE in multiple views to evaluate the position and stability of the device, its proximity to the AoV and the presence of significant TR. Before detaching the device, a hand injection in the ascending aorta through the guiding catheter was mandatory to document absence of LD interference with the AoV. Device was then released by a contra-clockwise cable rotation and the final result was assessed by TEE.

After achieving femoral hemostasis, IV heparin (starting dose of 25 units/kg/h) was administrated until the next morning, to maintain activated partial thromboplastin time 2–3 times greater than the reference value. Patients stayed in the hospital for overnight observation with vital signs monitoring. Platelet anti-aggregation therapy with oral aspirin 3–5 mg/kg/day (children) or 100 mg/day (adults) was prescribed for 6 months. The following day, all patients underwent clinical examination, and CXR to detect early complication such as occult hemorrhage and pulmonary complication. Twelve-lead EKG was performed to ensure sinus rhythm. Echocardiograms were also performed to detect pericardial effusion, aortic insufficiency, tricuspid valve stenosis, or insufficiency, LV outflow tract obstruction, LV function, and degree of shunting through the device. Urine analysis was done to rule out hemolysis in case of important RS or dark-colored urine. All patients in whom the procedure was uncomplicated were discharged from hospital 24 h after procedure. Endocarditis prophylaxis was done for the first six months in all patients but prolonged thereafter when persisted RS was documented on TTE. Patients were also instructed to avoid strenuous activity for one month.

### Follow-Up Protocol

Routine follow**-**up clinic visits were scheduled for 1 week then 1, 3, 6, and 12 months post-procedure and thereafter annually. New onset adverse events were monitored in each visit on the basis of basic clinical evaluation, TTE and EKG. The TTE included an assessment of changes in AR, TR, and RS. Holter monitoring (24 h) was performed only when clinically indicated.

### Statistical Analysis

Discrete variables were summarized as percentages and continuous variables as mean with standard deviation or median with range as appropriate. Statistical analysis of the categorical variables was conducted using Fisher's exact test and by ANOVA test for continuous variables. Statistical analyses were computed using the Statistical Package for the Social Sciences (SPSS Statistics), version 21 for Macintosh (IBM, Armonk, NY), with a *P* < 0.05 considered statistically significant. All reported *P* values are two-sided.

## Results

### Patient Characteristics (Table 1)

During the period of the study and following inclusion criteria, 51 patients (45.1% male) were identified. The mean age at the time of procedure was 7.4 ± 6.9 (range 0.3–33) years and the mean body weight was 25.4 ± 19.8 (range, 8–95) kg. There were 5 adults patients (age ≥ 18 years) (9.8%; female 80.0%). All 51 patients showed echocardiographic LV enlargement. Forty-nine patients showed aneurysmal type defect. Three patients (5.9%) had previously documented endocarditis and all of them were treated with antibiotics several months prior to attempted device closure. There was no residual vegetation along the margins of the defect at the time of the procedure. Three patients had minor associated CHD and were managed percutaneously in a different setting.

**Table 1:** Demographic and procedural characteristics.

	***n* = 51**
Age (years), *M ± SD (range)*	7.4 ± 6.9 (0.3–33)
Weight (kg), *M ± SD (range)*	25.4 ± 19.8 (8–95)
BSA (m^2^), *M ± SD (range)*	0.9 ± 0.4 (0.4–2.1)
Male, *N (%)*	23 (45.1)
Sub-aortic rim (mm), *M ± SD (range)*	4.7 ± 3.4 (0–14)
LV entry (mm), *M ± SD (range)*	10.5 ± 4.0 (4–20)
RV exit (mm), *M ± SD (range)*	4.7 ± 1.7 (2–8)
Indication for Closure^*^	
Left chamber enlargement	51 (100)
Endocarditis	3 (5.9)
**Associated CHD**	
Patent ductus arteriosus	1 (2.0)
Atrial septal defect	1 (2.0)
Muscular ventricular septal defect	1 (2.0)
**Device type**, ***N*** **(%)**	
Amplatzer Muscular VSD Occluder (AMO)	17 (33.3)
Amplatzer Duct Occluder (ADO)	7 (13.7)
Amplatzer Duct Occluder II (ADO II)	27 (52.9)
**Device delivery approach**, ***N*** **(%)**	
Venous	24 (47.1)
Arterial	27 (52.9)
Total procedural time, sheath in-out (min), *M ± SD (range)*	68.8 ± 33.6 (30–225)
Fluoroscopy time (min), *M ± SD (range)*	18.1 ± 12.9 (3.6–65.4)
Total dose area product (Gy.cm^2^), *M ± SD (range)*	34.0 ± 44.6 (1.1–244.8)
K_ar_ (mGy), *M ± SD (range)*	377.2 ± 365.5 (19–1,878)

*M ± SD = Mean ± Standard deviation. BSA, body surface area; LV, left ventricle; RV, right ventricle; CHD, congenital heart defects; K_ar_, Cumulative air kerma at the patient entrance reference point*.

* *More than one choice applied*.

### Procedural Characteristics, Outcomes, and Complications (Tables 1–3)

Device closure of the pmVSD was successful in 98% of the cases (50/51 patients) with the use of 52 Amplatzer devices. The only failure was attributed to inaccurate angiographic measurements misleading appropriate device size selection. In this patient, the initially chosen AMO device (size 6) pulled through the defect due to device's small size. The device was retrieved and the procedure was then aborted. In another patient's case, an (8 × 6) ADO device was judged suitable to close a pmVSD with no SAR, a 12 mm LV entry diameter and a 4 mm narrowest Doppler RV exit diameter. However, the unreleased device was small and unstable upon deployment which led to its replacement by an (10 × 8) ADO with a subsequent successful implantation.

**Table 2:** Procedural outcomes and complications.

**Successful implantation, *N (%), n = 51***	**50 (98.0)**
Transient CLBBB, *N (%), n = 51*	2 (3.9)
Device embolization, *N (%), n = 50*	1 (2.0)
Follow-up duration (days), *Median (range), n = 49*	194 (60–895)
**Persistent complications at the latest follow-up**, ***n = 49***	
CAVB, *N (%)*	1 (2.0)
Trivial residual shunt, *N (%)*	5 (10.2)
Valvular disturbances, *N (%)*	
Mild tricuspid regurgitation	5 (10.2)
Trivial aortic regurgitation	3 (6.1)

*CLBBB, complete left bundle branch block; CAVB, complete atrioventricular conduction block*.

**Table 3:** Groups comparison.

	**AMO, *n* = 17**	**ADO, *n* = 7**	**ADO II, *n* = 27**		
	***N*** **(%)**			**F^a^**	***p*-value**
Trivial residual shunt	3 (60.0)	–	2 (40.0)	1.680	0.389
Valvular disturbances	2 (25.0)	2 (25.0)	4 (50.0)	1.654	0.420
Mild tricuspid regurgitation	2 (40.0)	–	3 (60.0)	3.811	0.286
Trivial aortic regurgitation	–	2 (66.7)	1 (33.3)	–	–
	**M ± SD**			**F**^b^	***p*****-value**
Total procedural time, sheath in-out (min)	74.4 ± 30.8	102.9 ± 55.0	56.0 ± 18.9	7.191	**0.002**
Fluoroscopy time (min)	21.7 ± 9.0	30.7 ± 17.8	12.1 ± 10.1	9.170	**<0.001**
Total dose area product (Gy.cm^2^)	37.6 ± 21.2	125.9 ± 104.4	18.6 ± 17.8	13.911	**<0.001**
K_ar_ (mGy)	450.5 ± 258.9	1131.3 ± 659.4	231.0 ± 170.7	16.145	**<0.001**

a Fisher Test;

b *Anova*.

*M ± SD = Mean ± Standard deviation*.

*AMO, Amplatzer Muscular VSD Occluder; ADO, Amplatzer Duct Occluder; ADO II, Amplatzer Duct Occluder II*.

*K_ar_, Cumulative air kerma at the patient entrance reference point*.

*Bold values are significant p-values*.

A total of 50 Amplatzer devices were implanted as follows: 27 ADOII devices (54.0%), 16 AMO devices (32.0%), and 7 ADO devices (14.0%). The most commonly used ADOII device size was 6 × 4 (in 14 patients; 51.8%) followed by 5 × 4 and 4 × 4 (each one used in 6 patients; 22.2%), and 3 × 4 (in 1 patients; 3.8%). The most commonly used AMO device size was 6 (in 5 patients; 31.2%), and 8 (in 6 patients; 37.5%) followed by device size 10 (in 4 patients; 25.0%), and 14 (in one patient; 6.3%). The most commonly used ADO device size was 12 × 10 (in 4 patients; 57.1%), followed by device size 10 × 8 (in one patient; 14.3%), and 8 × 6 (in one patient; 14.3%), and 6 × 4 (in one patient; 14.3%). The mean total procedural time was 68.8 (± 33.6) min while the mean fluoroscopy time (FT) was 18.1 (± 12.84) min. All ADO II devices were delivered with the least amount of time (*p* = 0.002) and the lowest radiation exposure (*p* < 0.001) when compared to AMO and ADOI.

**Figure 1:**
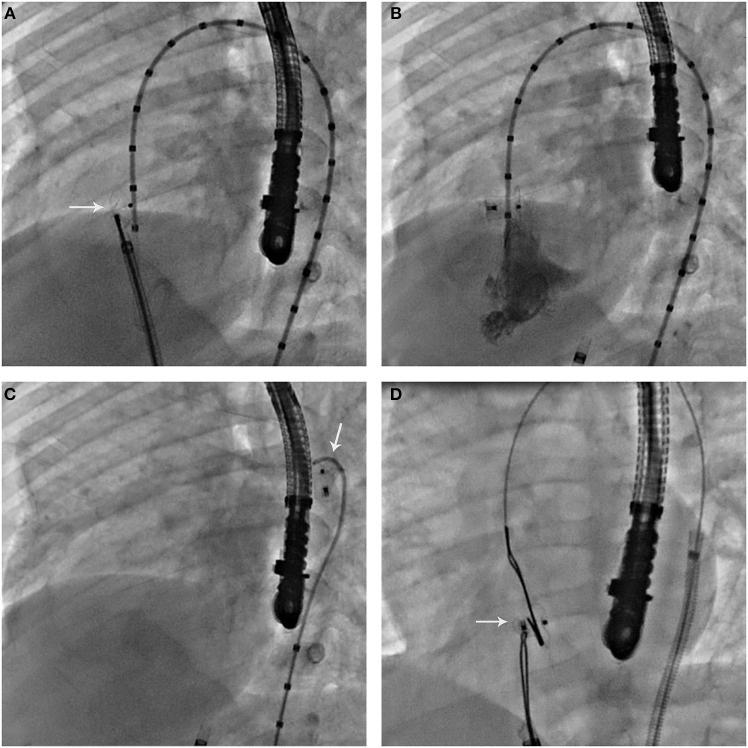
**(A)** Left ventricular angiography in 55–60° left anterior oblique to 20° cranial projection incidence, showing the unreleased ADO in good position within the defect. Note the misdiagnosed entrapment of the pigtail catheter between the device retention disk and the interventricular septum. **(B)** After releasing the device, left ventricular angiography in in 55–60° left anterior oblique to 20° cranial projection incidence, showing pigtail catheter entrapment with a satisfactory device position. **(C)** Pigtail catheter retrieval leading to accidental device displacement. Note the embolized device in the thoracic aorta. **(D)** Bilateral snaring of the embolized device after re-establishment of arteriovenous circuit for transvenous retrieval. Note the entrapped device across the defect with a satisfying position. Failure to unsnare it in this position or to pull it back across the defect for transvenous retrieval lead to surgical recapture from the iliac artery.

There was no procedure related mortality nor major vascular access complications. In one patient, a defect with 10 mm LV opening diameter, 7.5 mm depth and a 4.5 mm RV exit diameter was successfully closed with an ADO (8 × 6). However, following the completion of left ventriculography to check to check device position, we did not notice that the pigtail catheter was trapped between the device and the interventricular septum (IVS) leading upon its retrieval from the LV to device accidental embolization into the thoracic aorta. The device was surgically recaptured from the left iliac artery after multiple failed attempts to retrieve it transvenously (Figures 1A–D). In 2 other patients, AVC misconstruction led to transient severe bradycardia that was bailed out with circuit re-establishment leading to prolonged procedural duration.

**CHART 1:**
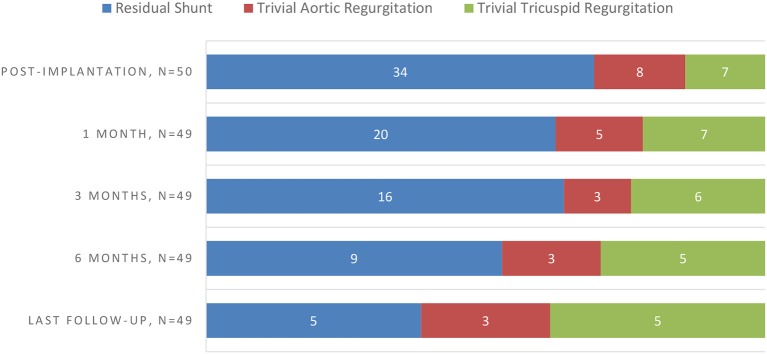
Progression of new-onset complications on follow-up (based on echocardiography).

On a median follow up period of 194 days (range, 60–895 days), 8/49 (16.3%) had persistent new-onset valvular disturbances, including 5 (10.2%) insignificant TR (AMO group, *n* = 2 and ADOII group, *n* = 3) and 3 (6.1%) trivial AR (ADOII group, *n* = 1 and ADO group, *n* = 2) (Table 3). All valvular lesions were considered as minor complications since none progressed in severity on follow-up nor needed to be cured until this manuscript was drafted. Complete occlusion rate was 32% (16/50) immediately upon completion of the procedure, rising up to 89.8% (44/49) at 6 months of follow-up. Persistent RS were trivial in all five cases (ADOII group, *n* = 2 and AMO group, *n* = 3) and presented benign courses with no hemodynamic significance and no incidence of mechanical hemolysis. Progression of new onset complications during follow-up (based on echocardiography) is summarized in Chart 1.

The most serious complication was CAVB and occurred in one 15 years old patient (2%) immediately upon release of an (12 × 10) ADO device. The implanted device was not retrieved since sinus rhythm was restored 5 min after IV atropine and steroids therapy, and the complication was classified as transient. After hospital discharge, this same patient was rediagnosed, on the 18 months routine follow-up EKG, with a persistent CAVB. She was then treated with permanent pacemaker and there was no sign of sinus rhythm recovery during follow-up visits. No future complication was encountered.

During the study period, no new other cases of CAVB or device surgical retrieval occurred and no major adverse event such as device embolization or malposition, thrombus or clot formation and thromboembolism were detected. None of the patients with a RS developed hemolysis. To this date, there has been no incidence of device related infectious endocarditis. We have seen that enlarged LV and LA decreased to normal size during the follow-up in all the patients even those with RS.

## Discussion

Perimembranous VSD is one of the most common type of CHD with recent growing interest in whether interventional approach can replace traditional open-heart surgical closure as the contemporary standard therapy for pmVSD (6–8). This new alternative has been wildly used in developing countries. Previously cited reports showed a variety of devices that have been used to treat pmVSD with promising results (4, 5). Despite that, percutaneous VSD closure is still not currently approved in the United States because of unacceptably high rates of post-procedural and late-onset heart block (HB) (10, 13–15).

In fact, CAVB is the huge cornerstone that limits the widespread use of transcatheter pmVSD closure with high reported incidence in young patients (13, 16) and no available clear data on the precise mechanisms involved in its occurrence (11, 17). Compared to surgery, in which CAVB usually appears immediately after the operation (10), reports showed that CAVB can occur at any time from a few minutes to months and years even after successful and uncomplicated procedures (12, 14, 18–20), may be reversible with medication or may become persistent, requiring permanent pacing (19, 21) when sudden death is escaped. Previous reports also showed that the Hiss bundle passes at the postero-inferior margin of pmVSD and is vulnerable to HB during device closure, especially in oversized device cases (22, 23). With this in mind, we believe that ADO II has an advantage as it may keep CAVB incidence low. Due to its flexible profile and small delivery sheath, ADOII can be deployed with easier manipulation through angulation and faster implantation process. Besides, ADO II is made of soft, fabric-free, multi-layered Nitinol wire mesh with low-profile retention disks, minimizing clamp force to the IVS and radial stress on the conduction system (24). This device property was previously emphasized by Vijayalakshmi et al. where none of the patients had HB (25).

**Figure 2:**
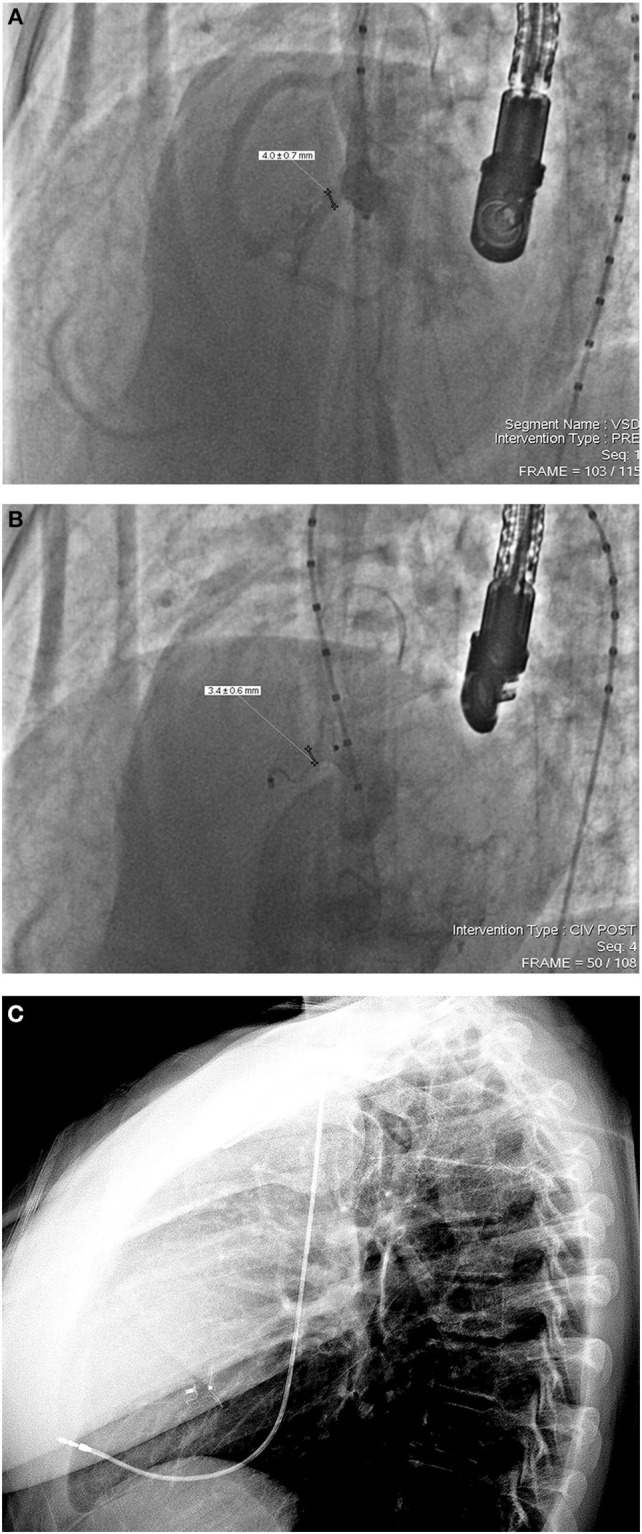
**(A)** Left ventricular angiography in in 55–60° left anterior oblique to 20° cranial projection incidence, showing an aneurysmal type pmVSD with a 4 mm right ventricular exist, 14 mm left ventricular entry and a 4 mm sub-aortic rim. **(B)** Note the compressed 12 × 10 ADO device inside the defect with a “bone-shaped” deformation immediately after release. **(C)** Lateral chest X-ray done the day following permanent pacing (18 months after device implantation). Note the device regaining its original conical shape despite tissue compression.

In our series, only one 15 year old patient developed transient CAVB immediately after the release of an ADO device. On the 18 months routine follow-up, this same patient was re-diagnosed with persistent CAVB requiring permanent pacing. This incidence confirms that HB can appear as a late unpredictable complication that requires high vigilance for appropriate diagnosis and treatment (26). Moreover, CAVB rate in our study was comparable with the one of surgical closure (27–33). In other words, device PmVSD closure using Amplatzer occluders may be a good alternative therapy to surgical closure in suitable patients. Ghaderian et al. reported that ADO with it shorter distal rim and no proximal disc reduces CAVB rates with less squeezing on the His bundle (34). For that, we retrospectively investigated this complication and found out that it could have been prevented. In fact, the chosen (12 × 10) ADO device for the closure of a tubular shaped defect (14 mm LV entry diameter, 4 mm exist diameter) was oversized and led to IVS compression (Figures 2A–C). We also believe that the immediate manifestation of our HB case was the consequence of a significant direct mechanical damage caused by the delivery system or by device deployment, while its late manifestation was highly due to fibrosis, compression or inflammation of the conduction system.

Aortic insufficiency is another serious complication to be aware of upon procedure completion and during follow-up. Upon full device deployment and before device release, TEE as well as dye injections were regularly performed to document LD non-interference with the AoV cusps. Despite this, we did face three cases of trivial AR that appeared upon device release, with no requiring therapy. For that, we believe that all our cases of AR were related to complex manipulation processes and difficult AVC establishment. This theory was supported by Zhao et al. who emphasized ADOII little effect on the AoV (33) and by other authors who discussed the ability of this device to adapt to different shapes and to fit into the defect without disturbing the AoV (24, 35–37).

Another important factor that greatly impacted the success of the intervention was the SAR length, with various studies suggesting a minimum length for safe deployment (35, 36). We carefully selected our devices, in defects with insufficient SAR, so that the LD could be safely deployed inside the defect left entry, aiming for less AR and more stability. On top of this, device selection was influenced by the aneurysmal anatomy, found in 96.1% of cases. In accordance with other authors, we found that implantation of patent arterial duct occluders would be more convenient in aneurysmal defects, since the retention disc can be set entirely within the aneurysm and the cylindrical portion of the device secures in an opening of the aneurysm on the RV side (14, 38, 39). Therefore, device will not get in contact with the AoV and will create minimal pressure on the IVS.

We did face five cases of insignificant TR of which none was documented after ADO implantation, as expected. With the absence of RD, septal leaflet will not be caught within the device, but given that aneurysm is often adjacent to or even part of the TV apparatus, caution should be always taken when the operator passes the wire and catheter through the valve to establish the AVC (6). Besides, a recent study reported late ADO II-related TR (40). However, we noticed that ADO II (with its short waist), when compared to AMO (with its larger lateral disk), has less chance to interfere with TV or to obstruct RV outflow tract.

In our experience, results of transcatheter VSD closure with Amplatzer occluders were satisfactory: the procedure was successfully performed in 98% of cases, confirming the results reported in other published studies (4, 5). However, closure rate was only 89.8% at six months of follow-up when compared to higher previous reports (36, 37, 41). In fact, some procedure related complications are reduced with precise defect sizing and proper device selection (42, 43). While oversized devices cause more damage to the adjacent structures, undersized devices may increase the rate of device embolization and RS. Respect to our device selection protocol helped us to control the risk of incomplete closure, yet 5 cases of RS where still encountered. In these patients with large aneurysm and multiple exists, we believe that incomplete occlusion occurred since the chosen device was unable to cover the defect LV entry and the aneurysm together, leading to para-prosthetic RS (5, 35). For that, further clinical experience will help us develop better algorithms using a combination of ultrasound and angiographic measurements of defect size and in choosing the correct device.

One case of device embolization occurred with ADO and was strictly accidental (Figures 1A–D). The incidence was transient and the patient had no sequelae. Retrospectively, this complication affected our device selection priority since we had the tendency to prioritize AMO over ADO, when ADOII was not applicable. The double disk design was more reassuring against the risk of embolization. This same accidental embolization was described by Muthusamy, who switched from using ADO to AMO for pmVSD closure, while emphasizing on retrograde approach advantages (44). Among few reporting off label-use of AMO for pmVSD closure with promising results (10, 14, 15), Muthusamy was also the only one recently discussing AMO limited profile (44). Although we do support his findings (45, 46) and believe that AMO large and stiff lateral disks presents high radial and clamping force tension on the IVS, 16 AMO devices were implanted in our series since we thought that the 7 mm relatively long waist might reduce clamping force, thereby minimizing injury to the conducting system.

Finally, an ideal device would perfectly occlude the defect and the aneurysm together, without damaging the surrounding valve and conduction tissues. Among available Amplatzer devices, ADO II (whenever applicable) can satisfy these conditions when high operator's experience and implantation techniques are met. Besides its soft and flexible design, the efficient retrograde deliverability is by far its biggest advantage (35, 36, 47). In fact, we found out that excessive shearing force on the defect margins and surrounding valves during AVC establishment may be higher than the ones in the retrograde approach, with significantly longer fluoroscopic time. Besides, while El Sisi et al emphasized on deploying LD at first (48), we noticed that RD deployment before proceeding with the rest of the device, allows its complete positional adjustment and lowers TR incidences.

## Study Limitations and Strengths

First and foremost, this was a single-center retrospective study with a limited number of participants and a wide age range. However, the strict protocol of percutaneous PmVSD closure in our institute before, during and after the procedure made the collected data comprehensive and accurate. In addition, all procedures were performed by the same operator, offering to a higher representation of routine practice. Besides the limited follow-up duration, this series of patients may not be representative of those encountered in developed countries. Well-designed prospective cohort studies that stratify patients based on age and device type are definitely needed to establish clinical guidelines, recommending routine pmVSD transcatheter closure.

## Conclusion

Percutaneous closure of pmVSD is a challenging and risky procedure, owing to variable anatomical morphology, proximity to valves and conduction tissues as well as complex manipulation process. The major key to improve the results of this treatment, while minimizing complications, consists in careful case and device selections as well as accurate defect sizing strategy. We showed that the mid-term results of our interventional approach for pmVSD closure using different Amplatzer occluders are equally promising with zero mortality and tolerable rate of morbidity. The procedure is relatively safe and effective. It appears that ADOII is the best available device to close defects with a diameter up to 5.5 mm, especially in aneurysmal type and in small children, because of its better profile and avoidance of a continuous AVC. Finally, CAVB remains the most potential serious complication that can occur during the procedure or any time later. For that, long-term follow-up in a large number of patients is mandatory to confirm safety of this intervention while monitoring unknown late onset complications.

## Data Availability

The raw data supporting the conclusions of this manuscript will be made available by the authors, without undue reservation to any qualified researcher.

## Ethics Statement

This study was reviewed and approved by Saint Joseph university research ethics committee.

## Author Contributions

RH performed calculations, analyzed the data, interpreted the results, and took the lead in writing the manuscript. ZS conceived of the presented idea and supervised the project. All authors discussed the results, read, and approved the final manuscript.

### Conflict of Interest Statement

The authors declare that the research was conducted in the absence of any commercial or financial relationships that could be construed as a potential conflict of interest.
